# Porcine TRIM21 Enhances Porcine Circovirus 2 Infection and Host Immune Responses, But Inhibits Apoptosis of PCV2-Infected Cells

**DOI:** 10.3390/v14010156

**Published:** 2022-01-15

**Authors:** Lin Yang, Xiaohua Liu, Liying Zhang, Xue Li, Xinwei Zhang, Guoyu Niu, Weilong Ji, Si Chen, Hongsheng Ouyang, Linzhu Ren

**Affiliations:** College of Animal Sciences, Key Lab for Zoonoses Research, Ministry of Education, Jilin University, 5333 Xi’an Road, Changchun 130062, China; linyang20@mails.jlu.edu.cn (L.Y.); lxh18@mails.jlu.edu.cn (X.L.); zhangliy@jlu.edu.cn (L.Z.); lixue9915@mails.jlu.edu.cn (X.L.); xwzhang17@mails.jlu.edu.cn (X.Z.); niugy9916@mails.jlu.edu.cn (G.N.); jiwl19@mails.jlu.edu.cn (W.J.); sichen20@mails.jlu.edu.cn (S.C.); ouyh@jlu.edu.cn (H.O.)

**Keywords:** tripartite motif protein 21 (TRIM21), porcine circovirus 2 (PCV2), immune response

## Abstract

Tripartite motif protein 21 (TRIM21) is an interferon-inducible E3 ligase, containing one RING finger domain, one B-box motif, one coiled-coil domain at the N-terminal, as well as one PRY domain and one SPRY domain at the C-terminal. TRIM21 is expressed in many tissues and plays an important role in systemic autoimmunity. However, TRIM21 plays different roles in different virus infections. In this study, we evaluate the relationship between porcine TRIM21 and PCV2 infection as well as host immune responses. We found that PCV2 infection modulated the expression of porcine TRIM21. TRIM21 can enhance interferons and proinflammatory factors and decrease cellular apoptosis in PCV2-infected cells. These results indicate that porcine TRIM21 plays a critical role in enhancing PCV2 infection, which is a promising target for controlling and developing the treatment of PCV2 infection.

## 1. Introduction

Porcine circovirus 2 (PCV2) is the pathogen causing porcine circovirus diseases and porcine circovirus-associated diseases (PCVD/PCVAD), which are prevalent in almost all pig farms worldwide and considered one of the most important infectious diseases causing immunosuppression in pigs. During infection, PCV2 interacts with the host proteins and replicates with the host component [1,2]. Notably, PCV2 infection promoted IFN-β production via activating the cGAS/STING signaling, while IFN-α, IFN-β, IFN-γ, concanavalin A (ConA), and IL-2 can enhance PCV2 replication [3,4,5,6,7,8,9,10,11]. On the other hand, intracellular host restriction factors, such as 3-hydroxy-3-methylglutaryl-coenzyme A reductase (HMGCR), complement component 1Q subcomponent-binding protein (C1QBP), and tyrosine 3-monooxygenase/tryptophan 5-monooxygenase activation protein (14-3-3β/α, YWHAB), play important roles in resisting virus infection [1,2,12,13,14]. Among the intracellular host factors, tripartite motif proteins (TRIMs) are a kind of newly discovered host protein involved in various cellular functions, including cell cycles, immunity, carcinogenesis, apoptosis, as well as virus infection [15,16,17].

TRIMs are one of the largest groups of E3 ubiquitin ligases, containing a RING-finger (RING) domain, one or two B-box motifs, and a coiled-coil (CC) domain (RBCC) at the N-terminal [18,19,20,21]. The RING domain has E3 ubiquitin ligase activity, which combines with E2 ubiquitin-binding enzyme, and then performs ubiquitination modification on the target protein. The B-box domain exists only in TRIM protein and is a cysteine-histidine-zinc finger motif, which mainly mediates the oligomerization of homologous or heterologous protein, promoting the formation of macromolecular and subcellular location. The CC domain is composed of multiple α-helices, which play a decisive role in the formation of the TRIM protein [20,21]. The C-terminal domain of the TRIM protein is a hypervariable region, which is the main domain of protein–protein interaction, and most TRIM proteins have a PRY/SPRY domain at the C-terminal [20,21].

Studies have shown that many TRIM proteins, especially human TRIMs, play an important role during virus infection. Most TRIMs restrict virus infection through different mechanisms, but a few TRIM proteins can promote virus infection by regulating innate immune response or directly interacting with viral proteins [18,20,21]. At present, there are few studies on porcine TRIMs participating in virus infection and regulating the immune response. For example, it was reported that the nuclear localization signal of porcine TRIM22 plays a key role in inhibiting type 2 porcine reproductive and respiratory syndrome virus (PRRSV) replication [22], and porcine TRIM21 RING-finger E3 ubiquitin ligase is critical for anti-PRRSV activity [15]. TRIM21 inhibits porcine epidemic diarrhea virus replication by proteasomal degradation of the viral nucleocapsid protein [23], while TRIM21 suppresses foot-and-mouth disease virus (FMDV) infection via specific antibody-mediated intracellular neutralization [24]. However, the influence of porcine TRIMs on PCV2 infection has not been reported.

In this study, the relationship between porcine TRIM21 and PCV2, and the mechanism of its effect on PCV2 proliferation, were evaluated, as well as the influence of interferons and pro-inflammatory factors.

## 2. Materials and Methods

### 2.1. Cells and Virus

Porcine kidney cell 15 (PK-15) and HEK293T cells were purchased from ATCC previously and stored in our lab [2]. Cells were cultured in DMEM (HyClone, Thermo Scientific, Waltham, MA, USA) containing 10% FBS (CLARK Bioscience, Webster, TX, USA) at 37 °C, 5% CO_2_ atmosphere, as described by Ouyang et al. [2].

PCV2 CC12 (GenBank No.: JQ955679) was isolated previously and stored in our lab [25].

Cells were plated in 6-well plates at 37 °C for 12 h to reach 70–80% confluency, followed by infection with PCV2 at a multiplicity of infection (MOI) of 1 for 1 h. Then, cells were washed with PBS twice and cultured in fresh DMEM containing 10% FBS for the indicated time.

### 2.2. Drugs and Antibodies

Plasmids pIRES2-EGFP and PX330 were kindly provided by Prof. Zhanjun Li (Jilin University, China). Plasmid pEGFP-MNDAL-N1 was constructed previously in our lab [2].

FITC-labeled goat anti-rabbit IgG (H + L), HRP-labeled goat anti-mouse IgG (H + L), HRP-labeled goat anti-rabbit IgG (H + L), DAPI, Enhanced BCA Protein Assay Kit, BeyoECL Plus kit, Cell lysis buffer for Western and IP, and Caspase3/8/9 ELISA Kit were purchased from Beyotime (Shanghai, China). Cell Counting Kit-8 (CCK-8) was purchased from Bioster (Wuhan, China).

TRIM21 rabbit polyclonal antibody, mouse P53 monoclonal antibody, and mouse Bcl-2 polyclonal antibodies were purchased from Proteintech (Wuhan, China). Lipofectamine 3000 Transfection Reagent and PTI-MEM were from Thermo Scientific (USA). Porcine TNF-α and IL-6 ELISA Kits were purchased from Absin (Shanghai, China). Rabbit PCV2 Cap antibody was from Biorbyt (Wuhan, China), and the rabbit anti-PCV2 Cap was prepared previously in the lab (1:200) [26]. Mouse IFN-α antibody, rabbit IFN-β antibody, mouse IFN-γ antibody, and mouse MNDA antibody were purchased from Santa Cruz Biotechnology (Santa Cruz, CA, USA). TIANamp Virus DNA/RNA Kit, TRNzol-A^+^ Reagent, 2× *Taq* plus PCR Mix, and FastKing-RT SuperMix kit were from Tiangen (Beijing, China). Luna^®^ Universal qPCR Master Mix (SYBR Green) was from New England Biolabs (NEB, Ipswich, MA, USA).

### 2.3. Plasmid Construction

Total RNA was extracted in PK-15 cells or virus-infected cells using TRNzol-A^+^ Reagent (Tiangen, Beijing, China), reverse transcribed with Oligo dT using FastKing-RT SuperMix kit (Tiangen, Beijing, China) according to the manufacturer’s instructions. Then, porcine TRIM21 was divided into two fragments and amplified with primer pairs TRIM21-1F/TRIM21-1R and TRIM21-2F/TRIM21-2R using 2× Pfu PCR MasterMix (Tiangen, Beijing, China). The full-length open reading frame (ORF) of porcine TRIM21 was amplified with the primer pair TRIM21-1F/TRIM21-2R via overlap extension PCR using two fragments of porcine TRIM21 as templates and sub-cloned into pIRES2-EGFP with *Nhe* I and *Sal* I, resulting in a recombinant plasmid pIRES2-EGFP-TRIM21. Primers (Supplemental Table S1) were designed based on the porcine TRIM21 gene (GenBank No.: NM_0011636492) and synthesized by GenScript (Nanjing, China).

To construct sgRNA expression, plasmids targeting porcine TRIM21, sgRNAs (Supplemental Table S1), were designed based on the porcine TRIM21 gene (GenBank No.: NM_0011636492) via the CCTop-CRISPR/Cas9 target online predictor (https://cctop.cos.uni-heidelberg.de:8043/, accessed on 20 November 2019) [27]. Forward and reverse sgRNAs (5 μL of each sgRNA) were annealed in 10 μL of 2× standard *Taq* reaction buffer (NEB, USA) and linked into PX330 with *Bbs* I (NEB, USA), generating a recombinant plasmids PX330-TRIM21-gRNA1 and PX330-TRIM21-gRNA2.

The plasmids were identified by PCR, restriction endonuclease enzyme analysis, and sequencing.

### 2.4. RNA/DNA Extraction and Amplification

Total cellular RNA was extracted using TRNzol-A^+^ Reagent, Virus RNA, or DNA was obtained using the TIANamp Virus DNA/RNA Kit according to the manufacturer’s instructions.

Reverse transcription was performed using the FastKing-RT SuperMix kit according to the manufacturer’s instruction at 42 °C for 15 min, followed by 95 °C for 3 min, and stored at −20 °C or used immediately.

PCR was performed using 2× Taq plus PCR Mix and real-time PCR was conducted using Luna^®^ Universal qPCR Master Mix (SYBR Green) with the indicated primers and annealing temperature (Supplemental Table S1). GAPDH was used as an internal control for real-time PCR. Each experiment was repeated three times.

### 2.5. Construction of Overexpression and Knocking out Cells

Plasmid pIRES2-EGFP-TRIM21 was linearized with *Ase* I (NEB, USA). PX330-TRIM21-gRNA and linearized pIRES2-EGFP-TRIM21 were purified by ethanol precipitation method to a final concentration of 3000 ng/μL, respectively.

To construct a porcine TRIM21 overexpression cell, PK-15 cells were cultured in a 6-well plate at 37 °C, 5% CO_2_, for 12 h to reach an 80–90% confluency. Cells were transfected with 2.5 μg pIRES2-EGFP-TRIM21 or pIRES2-EGFP using Lipofectamine 3000 Transfection Reagent according to the manufacturer’s instructions. A total of 72 h later, cells were cultured in DMEM supplemented with 5% FBS for 48 h, followed by incubation with G418 (400–600 μg/mL) for 12 h. Cell clones were further cultured in DMEM supplemented with 10% FBS and G418 (400 μg/mL), and identified by PCR (TRIM21-OE-F/R), real-time PCR, and Western blot. The positive cell clone was designated as TRIM21-OE.

To construct a porcine TRIM21 knocking-out cell, PK-15 cells (1 × 10^5^) cultured in the plate were washed with PBS three times, digested with trypsin, centrifuged at 1000× *g* for 5 min, and re-suspended in 300 μL PTI-MEM. Then, cells were gently mixed with 30 μg PX330-TRIM21-gRNA1 or PX330-TRIM21-gRNA2, followed by electro-transfection at 300 V, 1 ms, three times (BTX ECM 2001, USA). Five minutes later, at room temperature, the cells were transferred into a 6-well plate for 12 h, and further cultured in fresh DMEM supplemented with 10% FBS at 37 °C, 5% CO_2_, for 48–72 h. A positive cell clone was identified by real-time PCR (with primers TRIM21-F/R), Western blot, and designated as TRIM21-KO1 or TRIM21-KO2, respectively.

### 2.6. MTS Assay

Cell viability was evaluated via MTS assay using a Cell Counting Kit-8. Briefly, cells were plated in a 96-well plate for 48 h, washed with PBS three times, and cultured in 100 μL fresh DMEM, followed by incubation with 10 μL CCK8 solution at 37 °C, 5% CO_2_, for 2 h. Thereafter, cells were examined using an ELx800 microplate reader (Bio-TEK, Winooski, VT, USA) 1 h later and the OD_450_ value was recorded.

### 2.7. Western Blot (WB)

Cells were washed with PBS twice, lysed in cell lysis buffer for Western and IP supplemented with PMSF (1:200) on ice for 5 min, followed by centrifugation at 4 °C, 12,000× *g*, for 10 min. The protein was quantified using an Enhanced BCA Protein Assay Kit. Then, total protein (25 μg) was mixed with 5× SDS loading buffer, heated at 100 °C for 5 min, separated by 12% SDS-PAGE gel, and electro-transferred onto PVDF membranes (Millipore, Burlington, MA, USA). After blocking with 5% skim milk in TBS-T buffer for 90 min, the membrane was incubated with the indicated primary antibody for 2 h at room temperature, washed three times with TBST-T. Thereafter, the membrane was blotted with the secondary antibody for 90 min at room temperature, followed by washing with TBST-T three times. Subsequently, the band was detected using a BeyoECL Plus kit according to the manufacturer’s instructions.

### 2.8. Indirect Immunofluorescence Assay (IFA)

Cells in a 6-well plate were infected with PCV2 for 72 h, washed with PBS three times, and fixed with 80% cold-acetone at −20 °C overnight. After washing with PBS three times, cells were incubated with Rabbit anti-PCV2 Cap (1:200) [26] at 37 °C for 3 h and washed with PBS three times. Then, cells were blotted with FITC-labeled goat anti-rabbit IgG (H + L, 1:1000) at 37 °C for 45 min and washed with PBS three times, followed by staining with DPAI (1:500) at 37 °C for 7 min and washing with PBS three times. Finally, cells were examined via an Eclipse TE2000-V (Nikon, Tokyo, Japan).

### 2.9. ELISA

Cells were infected with PCV2 for 48 h (for TNF-α) or 72 h (for IL-6), and levels of TNF-α or IL-6 were detected using porcine TNF-α or IL-6 ELISA Kit. Briefly, 50 μL sample was added into a TNF-α or IL-6 antibody-coated plate and incubated at 37 °C for 1 h, washed with PBS three times, and incubated with HRP-conjugated antibody at 37 °C for 1 h. After three washes with PBS, 100 μL TMB solution was added to the well at 37 °C for 10–30 min, and the reaction was stopped by 50 μL H_2_SO_4_ (2M). Thereafter, the sample was examined using an ELx800 microplate reader (Bio-TEK) with absorbance at OD_450_.

### 2.10. Caspase Activity Assay

Cells were infected with PCV2 for 72 h, collected, and centrifuged at 4 °C, 1000× *g*, for 5 min. Then, caspase 3/8/9 activities were evaluated using a Caspase3/8/9 ELISA Kit according to the manufacturer’s instructions. Samples were measured using an ELx800 microplate reader (Bio-TEK) with absorbance at OD_405_.

### 2.11. Statistical Analysis

Statistical analysis was performed using GraphPad Prism 5 (San Diego, CA, USA) with a one-way or two-way analysis of variance (ANOVA). The results are shown as the mean ± standard deviation (SD) of three independent experiments. A *p* < 0.05 indicates statistical significance.

## 3. Results

### 3.1. PCV2 Infection Modulated the Levels of Porcine TRIM21

**Figure 1:**
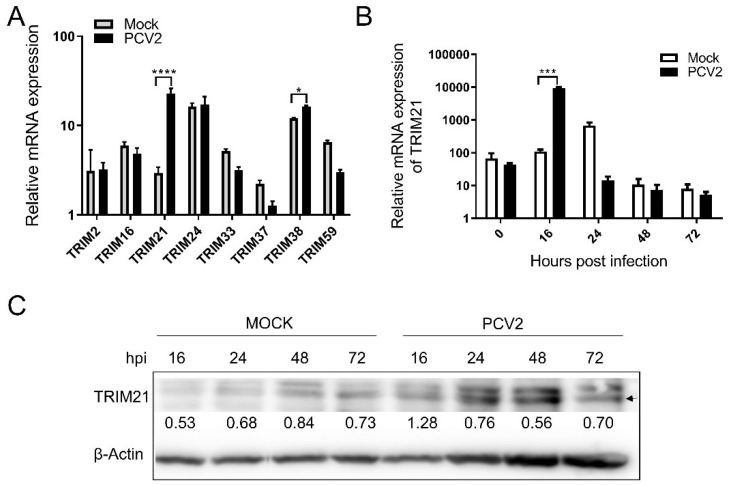
Expression of porcine TRIMs during PCV2 infection. (**A**) Expression of porcine TRIMs in PCV2-infected PK-15 cells. PK-15 cells were infected with PCV2 for 72 h, and total RNA and protein were collected and evaluated by qRT-PCR. The copies of TRIMs mRNA were normalized with GAPDH. (**B**,**C**) Expression of TRIM21 in PCV2-infected PK-15 cells. PK-15 cells were infected with PCV2, and total RNA and protein were collected at 0, 16, 24, 48, and 72 hpi, followed by qRT-PCR (**B**) and Western blot (**C**) analysis. The copies of TRIMs mRNA were normalized with GAPDH. For Western blot, TRIM21 rabbit polyclonal antibody (1:1000) and mouse anti-β-actin antibody (1:2000) were used as primary antibodies. HRP-labeled goat anti-rabbit IgG (H + L, 1:2000) and HRP-labeled goat anti-mouse IgG (H + L, 1:2000) were used as the secondary antibodies, respectively. The numbers indicate the relative protein levels of TRIM21 normalized by β-actin. Error bars indicate mean values of triplicates ± the standard deviations (SD). * *p* < 0.05; *** *p* < 0.001; **** *p* < 0.0001. Unprocessed original images can be found in Supplementary Figure S1.

We previously found by RNA-Seq analysis that several porcine TRIMs expressed significantly differently in PCV2-infected cells. Therefore, the expression levels of eight porcine TRIMs in PCV2-infected cells were further evaluated by qRT-PCR, including TRIM2, TRIM16, TRIM21, TRIM24, TRIM33, TRIM37, TRIM38, and TRIM59. As shown in Figure 1A, TRIM21 and TRIM38 were significantly upregulated in the virus-infected cells, while other TRIMs had no obvious increase, suggesting PCV2 infection can enhance the expression of TRIM21 and TRIM38. As human TRIM21 has been widely studied in antiviral activity, we chose porcine TRIM21 for further evaluation in this study. The research on porcine TRIM38 will be reported in another article later.

To further confirm the effect of PCV2 infection on porcine TRIM21, PK-15 cells in a 6-well plate were infected with PCV2, and total RNA and protein were collected at 0, 16, 24, 48, and 72 h post-infection (hpi), followed by qPCR. As shown in Figure 1B, the copies of porcine TRIM21 increased significantly at 16 hpi compared with that of the control group, which was further confirmed by Western blot analysis (Figure 1C). These results suggest PCV2 infection modulated the expression of porcine TRIM21.

### 3.2. Porcine TRIM21 Is Positively Associated with the PCV2 Infection

**Figure 2:**
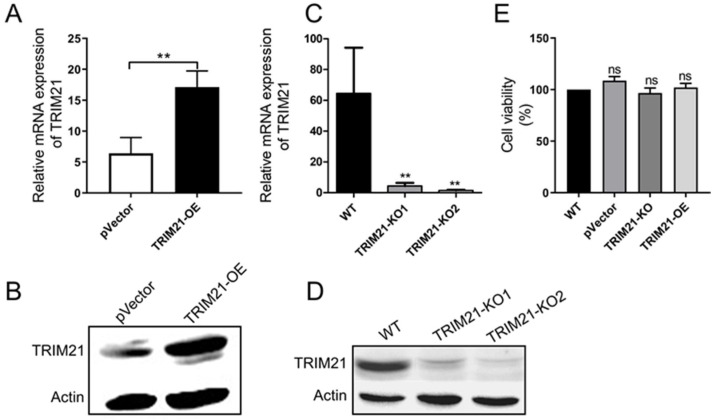
Construction of porcine TRIM21 overexpression and deletion cells. (**A**,**B**) TRIM21 overexpressed cells. PK-15 cells were transfected with pIRES2-TRIM21 and positive clones were identified by qRT-PCR (**A**) and Western blot (**B**). (**C**,**D**) TRIM21-deleted cells. PK-15 cells were transfected with PX330-TRIM21-gRNA, and positive clones were identified by qRT-PCR (**C**) and Western blot (**D**). TRIM21 rabbit polyclonal antibody (1:1000) and mouse anti-β-actin antibody (1:2000) were used as primary antibodies. HRP-labeled goat anti-rabbit IgG (H + L, 1:2000) and HRP-labeled goat anti-mouse IgG (H + L, 1:2000) were used as the secondary antibodies, respectively. (**E**) Cell viability. TRIM21-overexpression and -deleted cells were cultured and evaluated via MTS assay. Error bars indicate mean values of triplicates ± the standard deviations (SD). ** *p* < 0.01; ns, not significant. The copies of TRIM21 mRNA were normalized with GAPDH. Unprocessed original images can be found in Supplementary Figure S2.

To further examine the relationship between porcine TRIM21 and PCV2 infection, TRIM21 overexpression cells and TRIM21 knocking-out cells were constructed, designated as TRIM21-OE and TRIM21-KO, respectively. As shown in Figure 2A–D, levels of TRIM21 significantly increased in the TRIM21-OE cells compared with that of the control cells (Figure 2A,B), and the expression of TRIM21 in positive clone cells was inhibited by sgRNA1 or sgRNA2 targeting porcine TRIM21 (Figure 2C,D). Since the sgRNA2 exhibited a higher inhibitory effect than sgRNA1, TRIM21-KO2 cells were used in the following studies. Furthermore, the overexpression and knocking out of TRIM21 do not affect cell viability (Figure 2E).

**Figure 3:**
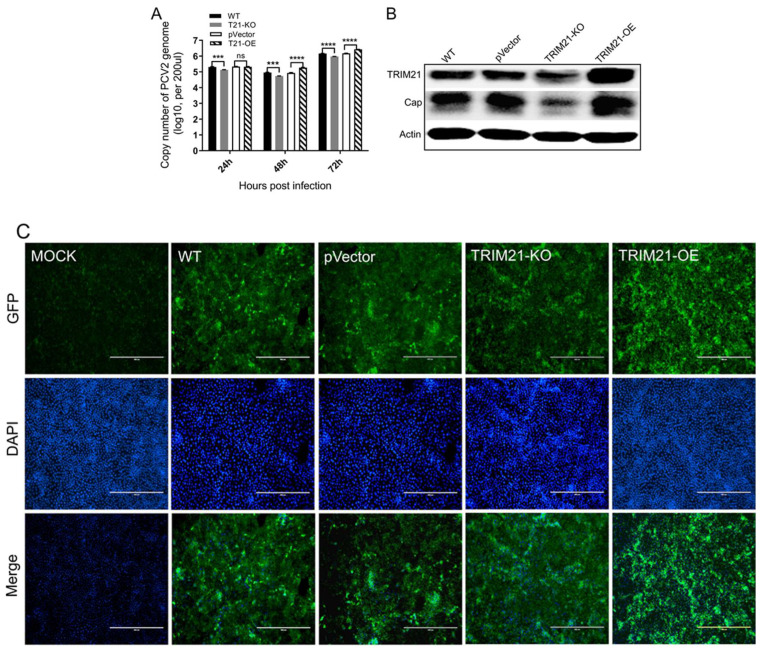
Porcine TRIM21 enhances PCV2 infection. (**A**) qRT-PCR. Cells were infected with PCV2 for 24, 48, or 72 hpi, and the copies of the PCV2 genome were evaluated by qRT-PCR. *** *p* < 0.001; **** *p* < 0.0001; ns, not significant. (**B**) Western blot. Cells were infected with PCV2 for 72 hpi, and the total protein was collected and analyzed by Western blot. TRIM21 rabbit polyclonal antibody (1:1000), rabbit PCV2 Cap antibody (1:500), and mouse anti-β-actin antibody (1:2000) were used as primary antibodies. HRP-labeled goat anti-rabbit IgG (H + L, 1:2000) and HRP-labeled goat anti-mouse IgG (H + L, 1:2000) were used as the secondary antibodies, respectively. Unprocessed original images can be found in Supplementary Figure S3. (**C**) IFA. Cells were infected with PCV2 for 48 hpi, fixed, and analyzed by IFA. Scale bar = 400 μm.

Thereafter, TRIM21-KO and TRIM21-OE were infected with PCV2 for 24, 48, or 72 h, and the infection of PCV2 was evaluated. As shown in Figure 3A, the copies of the PCV2 genome were significantly decreased in the TRIM21-KO cells compared with that of the WT group at 24, 48, or 72 hpi. Meanwhile, the copies of the PCV2 genome were increased obviously in the TRIM21-OE cells compared with that of the control (pVector) group at 48 and 72 hpi. These results were further confirmed by Western blot (Figure 3B) and IFA (Figure 3C), indicating that TRIM21 is positively associated with PCV2 infection, which may be involved in the virus infection.

### 3.3. Porcine TRIM21 Decreases Cellular Apoptosis in PCV2-Infected Cells

**Figure 4:**
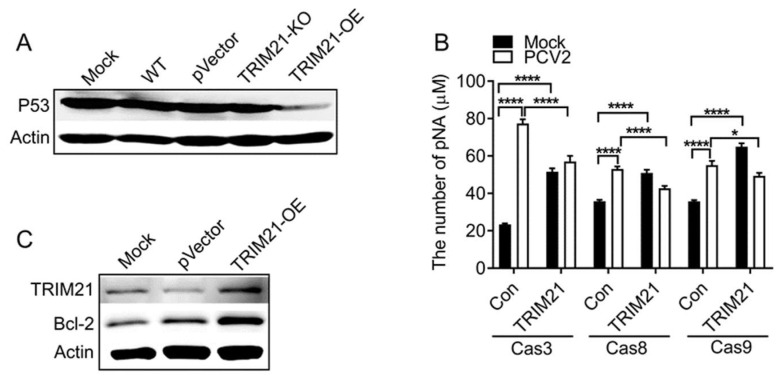
Porcine TRIM21 decreases cellular apoptosis in PCV2-infected cells. (**A**) p53. PK-15 cells were infected with PCV2 for 72 h, followed by Western blot. (**B**) Caspase 3, 8, and 9 activities. PK-15 cells (control cells, Con) and TRIM21-OE cells were infected with PCV2 for 72 h, followed by caspases activities analysis using the Caspase (3, 8, and 9) Activity Assay Kit. * *p* < 0.05; **** *p* < 0.0001. (**C**) Bcl-2. PK-15 cells were infected with PCV2 for 72 h, followed by Western blot. Mouse anti-P53 monoclonal antibody (1:1000), mouse anti-Bcl-2 polyclonal antibody (1:1000), and mouse anti-β-actin antibody (1:2000) were used as primary antibody, and HRP-labeled goat anti-mouse IgG (H + L, 1:2000) was used as the secondary antibody. Unprocessed original images can be found in Supplementary Figure S4.

We previously found that PCV2 infection induces cellular apoptosis [28]. Therefore, we examined the levels of p53 protein in PCV2-infected cells. The results showed that p53 was decreased in TRIM21-OE cells compared with that of the control group, while no significant difference was observed in TRIM21-KO cells compared with the control group (Figure 4A), suggesting porcine TRIM21 may inhibit PCV2-induced apoptosis. To further confirm this result, we investigated the levels of caspase 3, 8, and 9 in the TRIM21-overexpressed cells (TRIM21-OE) during PCV2 infection. As shown in Figure 4B, the levels of caspase 3, 8, and 9 were significantly inhibited in TRIM21-overexpressed cells compared to that of the control cells during PCV2 infection, which further confirmed the inhibitory effect of porcine TRIM21 on cellular apoptosis. Moreover, overexpression of TRIM21 can significantly increase the expression of Bcl-2, a regulator of cell death (apoptosis), in PCV2-infected cells (Figure 4C). These results indicate that porcine TRIM21 inhibits PCV2-induced apoptosis in PK-15 cells.

### 3.4. Porcine TRIM21 Enhances Interferons and Proinflammatory Factors in PCV2-Infected Cells

**Figure 5:**
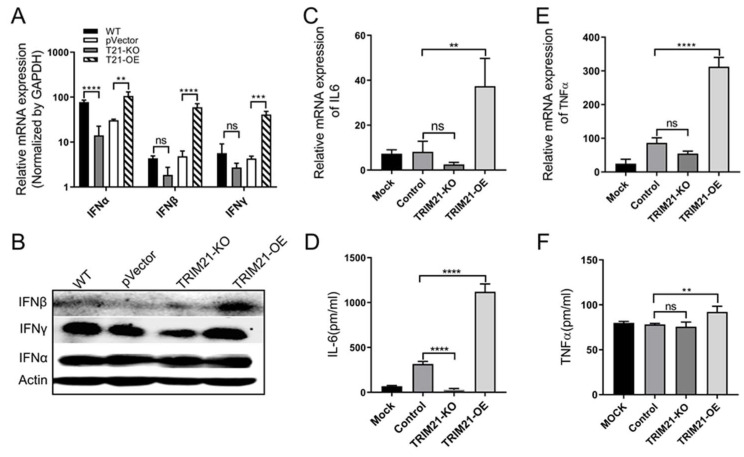
Porcine TRIM21 enhances interferons and proinflammatory factors in PCV2-infected cells. Error bars indicate mean values of triplicates ± the standard deviations (SD). ** *p* < 0.01; *** *p* < 0.001; **** *p* < 0.0001; ns, not significant. The copies of relative mRNA were normalized with GAPDH. (**A**,**B**) IFN. Cells were infected with PCV2 for 72 h, followed by qRT-PCR (**A**) and Western blot (**B**) assays. Mouse IFN-α antibody (1:400), rabbit IFN-β antibody (1:800), mouse IFN-γ antibody (1:500), and mouse anti-β-actin antibody (1:2000) were used as primary antibody, and HRP-labeled goat anti-mouse IgG (H + L, 1:2000) or HRP-labeled goat anti-rabbit IgG (H + L, 1:2000) were used as the secondary antibody. Unprocessed original images can be found in Supplementary Figure S5. (**C**,**D**) IL-6. Cells were infected with PCV2 for 72 h, followed by qRT-PCR (**C**) and ELISA (**D**) assays. (**E**,**F**) TNF-α. Cells were infected with PCV2 for 48 h, followed by qRT-PCR (**E**) and ELISA (**F**) assays.

Existing evidence showed that TRIM21 involves interferons (IFNs) and proinflammatory factors production [29,30,31]. Therefore, we evaluated levels of IFN-α, -β, -γ, IL-6, and TNF-α in PCV2-infected TRIM21-OE and TRIM21-KO cells. As shown in Figure 5A, IFN-α was significantly decreased in TRIM21-KO cells compared with that of the wild type group, whereas levels of IFN-α, -β, and -γ were increased in TRIM21-OE cells compared to that of the control group (pVector group). These results were further confirmed by Western blot, and the levels of IFN-β and IFN-γ increased significantly in the TRIM21-OE group compared to that of the pVector control group (Figure 5B). Meanwhile, the expression of IL-6 (Figure 5C,D) and TNF-α (Figure 5E,F) was significantly promoted in the TRIM21-OE cells compared to that of the control group.

These results demonstrate that TRIM21 overexpression enhances IFN-β, IFN-γ, IL-6, and TNF-α, suggesting TRIM21 has positive regulation on interferons and proinflammatory factors in PCV2-infected cells.

### 3.5. Porcine TRIM21 Increases Porcine MNDAL Expression

**Figure 6:**
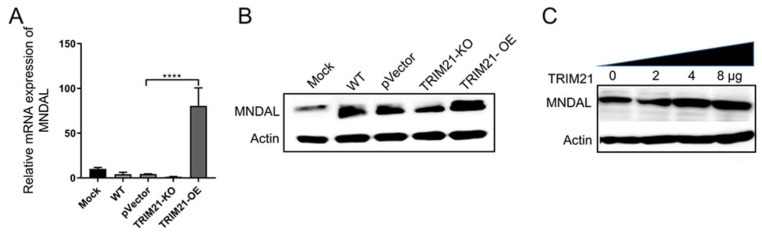
Porcine TRIM21 increases porcine MNDAL expression. (**A**,**B**) Cells were infected with PCV2 for 72 h, followed by qRT-PCR (**A**) and Western blot (**B**). (**C**) HEK293T cells in the 6-well plate were co-transfected with pIRES2-EGFP-TRIM21 (0, 2, 4, and 8 μg) and pEGFP-MNDAL-N1 (0.5 μg) for 48 h, followed by Western blot. Mouse MNDA antibody (1:500) and mouse anti-β-actin antibody (1:2000) was used as the primary antibody, HRP-labeled goat anti-mouse IgG (H + L, 1:2000) was used as the secondary antibody. **** *p* < 0.0001. The copies of MNDAL mRNA were normalized with GAPDH. Unprocessed original images can be found in Supplementary Figure S6.

Studies have shown that TRIM21 participates in STING-mediated negative feedback regulation of the DNA sensor IFI16, a member of the interferon inducible-200 (IFI200) family, which leads to the degradation of IFI16 via the ubiquitin–proteasome pathway, and thus balancing the overproduction of IFN-I caused by a virus infection [32]. We and other groups previously found that the porcine myeloid nuclear differentiation antigen-like (MNDAL), and its counterpart, the human HIN-200 cluster and mouse interferon inducible-200 (IFI200) family, are involved in virus infection, inflammation, and interferon response [33,34]. Therefore, we analyzed the effect of porcine TRIM21 on MNDAL expression during PCV2 infection. The results in Figure 6 demonstrated that overexpression of TRIM21 in PK-15 cells can significantly promote the levels of MNDAL in transcriptional level (Figure 6A) and translation level (Figure 6B), whereas no significant difference was found between the control groups and the knock-out group (TRIM21-KO).

Moreover, to further confirm the enhancement effect of porcine TRIM21 on porcine MNDAL, plasmids expressing porcine TRIM21 and MNDAL were co-transfected into human cell line HEK293T cells. The results in Figure 6C showed that the expression of porcine MNDAL in HEK293T cells can also be upregulated by porcine TRIM21 (pIRES2-EGFP-TRIM21), as the MNDAL levels of the latter two (4 and 8 μg) groups were higher than that of the other two groups (0 and 2 μg). In addition, the levels of MNDAL can be enhanced in both porcine cells (PK-15 cells, Figure 6A,B) and human cells (HEK293T cells, Figure 6C), which further demonstrated that porcine TRIM21 can increase the expression of MNDAL.

## 4. Discussion

TRIM21, also denoted as autoantigen Ro52, is expressed in many tissues, especially in T cells, macrophages, dendritic cells, and other leukocytes [29,35,36,37]. It is an interferon-inducible E3 ligase that can ubiquitinate IRF-3 and IRF-8 and enhance the expression of cytokines in macrophages [35,38,39]. Furthermore, TRIM21 can regulate the expression of IFN by feedback, but the mechanism of TRIM21 regulating IFN is different in different virus-infection processes. Higgs and colleagues found that TRIM21 negatively regulates IFN-β production by polyubiquitin-mediated degradation of IRF3 in Sendai virus- and Japanese encephalitis virus-infected cells [31,40], while TRIM21 can promote the production of IFN-β by enhancing the dimerization and phosphorylation of IRF3 in Coxsackievirus B3 (CBV3)-infected cells, thus restricting CBV3 replication [29]. Moreover, TRIM21 may modulate the activation or proliferation of T cells [37,41]. Wei et al. found that porcine TRIM21 was significantly upregulated in vitro and in vivo after porcine reproductive and respiratory syndrome virus infection (PRRSV) infection, and then inhibited the virus infection via the activity of RING-finger E3 ubiquitin ligase in porcine TRIM21 [15]. We analyzed 61 identified porcine TRIMs by bioinformatics and found that porcine TRIM21 consisted of 469 amino acids, with the RBCC domain at the N terminal and PRY/SPRY domain at the C-terminal, which are conserved among different species (data not shown). These results are consistent with the results reported by Fan et al., in that porcine TRIM21 has a high sequence similarity (>81%) at the amino acid level to cattle, feline, rhesus, and humans [24]. Furthermore, porcine TRIM21 is also expressed in various tissues, including the heart, liver, spleen, lung, kidney, and lymph nodes, and can also be detected throughout PK-15 cells, with a relatively higher level in the cytoplasm [24]. These results indicate that porcine TRIM21 plays important roles in systemic immunity (including physiological immune responses and pathological autoimmune responses) and virus infection [29,35,36,37]. In this study, we found that overexpression of porcine TRIM21 enhanced the production of IFNs, pro-inflammatory factors IL-6, TNF-α, and interferon inducible-200 (IFI200) protein MNDAL, while the knocking-out of porcine TRIM21 had the opposite effect. Moreover, as reported previously, IFN and IL-2 enhance PCV2 infection [3,4,5,6,7]. IL-10 promoted persistent infection of PCV2 and aggravated tissue damage by inhibiting T cell infiltration [42], which, in turn, leads to immunosuppression in persistent and chronic viral infection [42,43]. These results suggest porcine TRIM21 positively regulates immune responses during PCV2 infection, thus enhancing PCV2 replication.

In addition to regulating immune responses, TRIM21 can also interact with the virus directly or indirectly. As reported, human papillomavirus (HPV) oncoprotein E7 interacts with IFI16 and promotes the ubiquitin-mediated degradation of IFI16 by recruiting the E3 ligase TRIM21, resulting in the inhibition of cell pyroptosis during HPV infection [44]. TRIM21 inhibits Hepatitis B virus (HBV) DNA replication by ubiquitination of the viral DNA polymerase using its RING domain and SPRY domain [45]. PCV2 Cap can directly interact with cellular C1QBP, thus inhibiting the ubiquitin-mediated degradation of C1QBP; in turn, the increased stability of C1QBP enhances the phagocytic activity of porcine macrophages through the PI3K signaling pathway [46]. Moreover, we previously found that PCV2 infection was inhibited by HMGCR at the early stage of the infection, whereas being prompted by protein kinase C (PKC) at the late stage of the infection [12]. On the contrary, PCV2 inactivates HMGCR via the interaction of virus Rep and Cap proteins with HMGCR [2]. During the early stage of PCV2 infection, AMPK activity fluctuated in PCV2-infected cells [2]. Wang et al. reported that porcine Makorin RING finger protein 1 (pMKRN1) was upregulated in the early stage of PCV2 infection, and thus mediating the ubiquitination and degradation of viral Capsid to block the virus replication [47]. However, persistent PCV2 infection leads to the downregulation of pMKRN1, thus avoiding the degradation of viral Capsid and promoting virus replication and pathogenesis in its target tissue [47]. These results further confirmed that the interaction between a virus and host is a dynamic process, and the expression of host protein will change dynamically during the infection. In this study, we also found that porcine TRIM21 was significantly upregulated at 16 hpi in PCV2-infected cells, which, in turn, further promoted the proliferation of PCV2. PCV2 infection was significantly inhibited in the TRIM21-KO cells and enhanced in the TRIM21-OE cells compared with that of the control groups, indicating that TRIM21 is positively associated with PCV2 infection. Therefore, whether porcine TRIM21 can interact with PCV2 proteins remains to be evaluated. The exact mechanism of porcine TRIM21 regulating PCV2 infection is under study.

Apoptosis is an important strategy for the host to resist virus invasion. During PCV2 infection, phosphorylation of ASK1 activates the JNK and p38 pathway, thus resulting in apoptotic responses in the PCV2-infected cells [48]. Meanwhile, the phosphatidylinositol 3-kinase/Akt signaling pathway (PI3K/AKT) was also activated to negatively modulate the JNK and p38 MAPK pathway via ASK1 signaling, thereby inhibiting apoptosis, facilitating cell survival and viral replication [48,49]. Persistent PCV2 infection triggered unfolded protein reaction in PK-15 cells by selective activation of PKR-like endoplasmic reticulum (ER) kinase (PERK) via the PERK–eIF2α–ATF4–CHOP pathway [50], suggesting a relationship between ER stress and autophagic and apoptotic responses during PCV2 infection. We previously also found that HMGCR is negatively associated with PCV2-induced apoptosis [28]. Here, we found that porcine TRIM21 inhibited PCV2-induced apoptosis in PK-15 cells by increasing the expression of apoptosis inhibitor Bcl-2, which further confirms that porcine TRIM21 is positively corrected with PCV2 infection.

## 5. Conclusions

In the present study, we found that PCV2 infection modulated the expression of porcine TRIM21, which was positively associated with PCV2 infection, by enhancing immune responses and inhibiting cellular apoptosis. Our findings suggest that porcine TRIM21 plays a critical role in PCV2 infection, which may provide a potential target for the control of, and developing a treatment for, PCV2 infection.

## Data Availability

All data generated or analyzed during this study are included in this published article as Supplementary Materials.

## Supplementary Materials

The following are available online at https://www.mdpi.com/article/10.3390/v14010156/s1. Table S1: Sequence of primers and oligonucleotides; Figure S1: Original Western images used for preparing Figure 1C; Figure S2: Original Western images used for preparing Figure 2B,D; Figure S3: Original Western images used for preparing Figure 3B; Figure S4: Original Western images used for preparing Figure 4A,C; Figure S5: Original Western images used for preparing Figure 5A,B; Figure S6: Original Western images used for preparing Figure 6B,C.
