# Supplementary material for: Porcine TRIM21 Enhances Porcine Circovirus 2 Infection and Host Immune Responses, But Inhibits Apoptosis of PCV2-Infected Cells
Source: Viruses. 2022 Jan 15;14(1):156. doi: 10.3390/v14010156 (PMC8780438; doi:10.3390/v14010156)
Supplement: Supplementary file 1 [file viruses-14-00156-s001.zip › viruses-1536619-supplementary.pdf]

Porcine TRIM21 enhances PCV2 infection and immune responses but inhibits apoptosis in PCV2-infected cells

Lin Yang[#], Xiaohua Liu[#], Liying Zhang[#], Xue Li, Xinwei Zhang, Guoyu Niu, Weilong Ji, Si Chen, Hongsheng Ouyang, Linzhu Ren*

College of Animal Sciences, Key Lab for Zoonoses Research, Ministry of Education, Jilin University, 5333 Xi'an Road, Changchun 130062, China

Running title: Porcine TRIM21 increases PCV2 infection

✉ Correspondence: renlz@jlu.edu.cn; Tel.: +0086-431-87836175

* These authors contributed equally to this work.

This file includes:

Supplemental Table S1

Figures S1 to S6

Supplemental Table S1

Supplemental Table S1 Sequence of primers and oligonucleotides

Primer and oligonucleotide	Sequence (5'-3')	Length (bp)	Annealing temperature
TRIM2-F	TGACATCCATTCCACCTTTGA	111	60
TRIM2-R	GACTGCAGGACTTTGTGTTTG		
TRIM16-F	AGCGTCTACATCGGACTCAA	97	60
TRIM16-R	CCTTGTAATCCTGCAGCAACT		
TRIM21-F	CCGTCTCCTTCTACAACATCAG	121	60
TRIM21-R	GCATTTCTTCCACCGTCATTG		
TRIM24-F	ATACTTACGTCCCTGCTCTTG	196	60
TRIM24-R	GGTCGTCCTCTTTCCTTGT		
TRIM33-F	CAGCTCCTGGTTATACTCCTAATG	105	60
TRIM33-R	GAAGTCGAAGCTGTGCTAAGT		
TRIM37-F	GAGGGAGAACTCATGGAAGATG	115	60
TRIM37-R	TGGTAGCAGCAGAGCATAAG		
TRIM38-F	GGTGGGATTTAGGAGTCTGTATG	149	60
TRIM38-R	CCCTCAGTTGAAGGGAAGTTAG		
TRIM59-F	TCACCTGCCCTGAACATTAC	90	60
TRIM59-R	GGATGACCATGATGTTGACCTA		
TRIM21-1F	ATGGCCTCAGCACTGCCCTTGGCAAGGATG	753	56
TRIM21-1R	CTTTCCAGGACACTCTTCACCTCCTGTAG		
TRIM21-2F	GGTGAAGAGTGTCCTGGAAAGGAGTGAGTCTTGGAACCT	672	56
TRIM21-2R	CCAGCCCGTCCTCAGTGGACAGAGGGTCAGGGGGGGT		
PCV2-F (1)	TGTAGTATTCAAAGGGCACAGAGC	131	60
PCV2-R (1)	CGGATATACTATCAAGCGAACCAC		
TRIM21-sgRNA1*	Forward: CACCGAAGCTTCACCTGTTCTGTA<br>Reverse: AAACTACAGAACAGGTGAAGCTTC	24	
TRIM21-sgRNA2*	Forward: CACCGTCGGCAGGTGGCCAACATGG<br>Reverse: AAACCCATGTTGGCCACCTGCCGAC	24	
TRIM21-KO-F	CCTCACACAGCTCTTCTCATCGCCTTCTTTC	545	58
TRIM21-KO-R	GCCTGCTCGTGCCCAGGTGCCTCCGGGCT		
TRIM21-OE-F	GTAACAACTCCGCCCCATTGAC	399	58
TRIM21-OE-R	GCTTCTCTCCGTGCACCGCAC		
GAPDH-F	GCCATCACCATCTTCCAGG	437	60
GAPDH-R	TCACGCCCATCACAAACAT		
MNDAL-F	CTCTCCTGACACCCAGAAATG	90	60
MNDAL-R	CACTGTGGAAACCATCCTCT		
TNF-F	CACGTTGTAGCCAATGTCAAAG	136	60
TNF-R	GAAGAGGACCTGGGAGTAGAT		
IL-6-F	ACCTGGACTACCTCCAGAAA	111	60
IL-6-R	GGTGGCTTTGTCTGGATTCT		
IFNα-F	CATCCTGGCTGTGAGGAAATA	142	60
IFNα-R	CCTTCTTCCTGAGTCTGTCTTG		

IFNβ-F	GGAGACAATCCTGGAGGAAATC	104	60
IFNβ-R	TACTCCTTGGACTTCAGGTACT		
IFNγ-F	AGCCATCAGTGAACTCATCAA	81	60
IFNγ-R	CTCTCTGGCCTTGGAACATAG		

* Note: sgRNAs were incubated at 95 ℃ for 5 min, followed by slow cooling to room temperature.

Supplemental figures

Figure S1 Original Western images used for preparing Figure 1C.

TRIM21	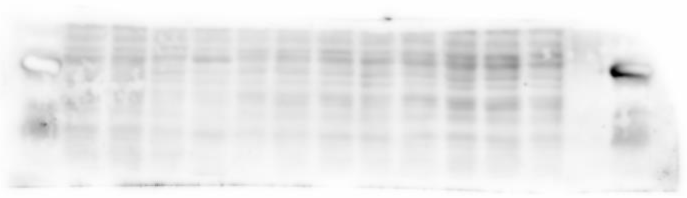
Actin	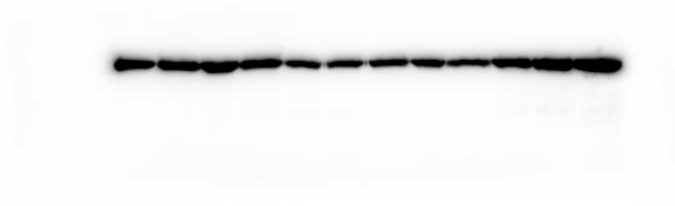

Figure S2 Original Western images used for preparing Figure 2B and 2D.

TRIM21-OE	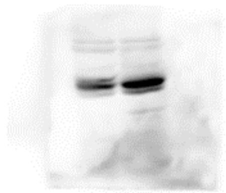
Actin	

TRIM21-KO	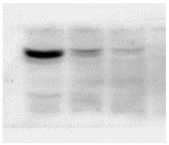
Actin	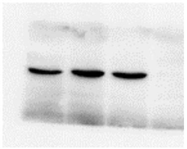

Figure S3 Original Western images used for preparing Figure 3B.

TRIM21	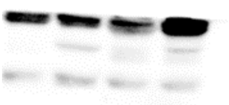
Cap	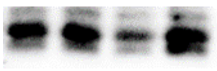

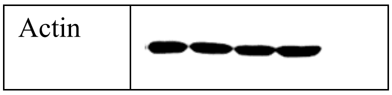

Figure S4 Original Western images used for preparing Figure 4A and 4C.

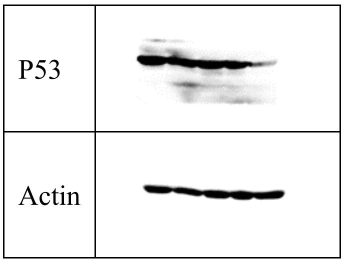

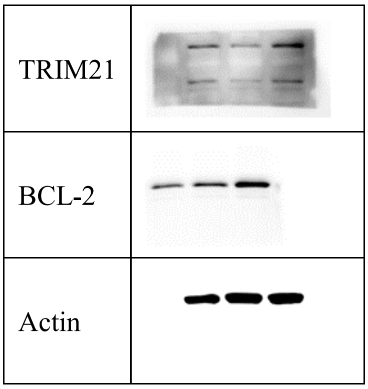

Figure S5 Original Western images used for preparing Figure 5A and 5B.

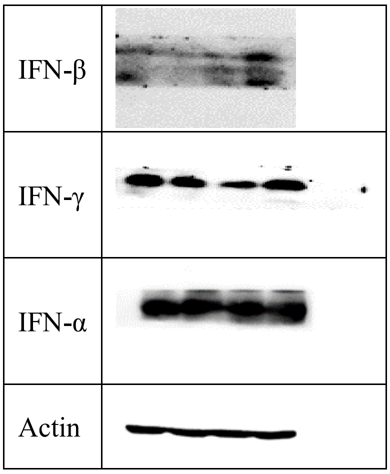

Figure S6 Original Western images used for preparing Figure 6B and 6C.

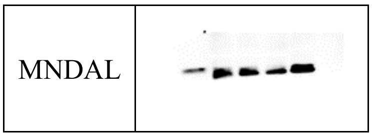

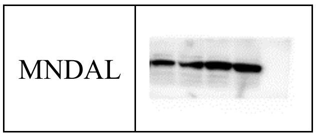

Actin

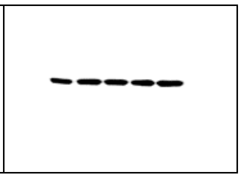

Actin

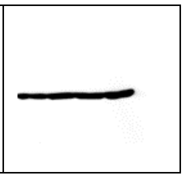

Reference

1. Liu X, Ouyang T, Ouyang H, et al. Human cells are permissive for the productive infection of porcine circovirus type 2 in vitro. Sci Rep 2019;9:5638.
